# Two Different Immune Profiles Are Identified in Sentinel Lymph Nodes of Early-Stage Breast Cancer

**DOI:** 10.3390/cancers16162881

**Published:** 2024-08-19

**Authors:** Joana Martins Ribeiro, João Mendes, Inês Gante, Margarida Figueiredo-Dias, Vânia Almeida, Ana Gomes, Fernando Jesus Regateiro, Frederico Soares Regateiro, Francisco Caramelo, Henriqueta Coimbra Silva

**Affiliations:** 1 Laboratory of Sequencing and Functional Genomics of UCGenomics, Faculty of Medicine, University of Coimbra, 3000-548 Coimbra, Portugal; 2 Institute for Clinical and Biomedical Research (iCBR), Centre of Investigation on Environment, Genetics and Oncobiology (CIMAGO), Faculty of Medicine, University of Coimbra, 3000-548 Coimbra, Portugal; 3 Gynecology Department, Coimbra Hospital and University Center, Unidade Local de Saúde de Coimbra, 3004-561 Coimbra, Portugal; 4 Gynecology University Clinic, Faculty of Medicine, University of Coimbra, 3000-548 Coimbra, Portugal; 5 Department of Pathology, Coimbra Hospital and University Center, Unidade Local de Saúde de Coimbra, 3004-561 Coimbra, Portugal; 6 Institute of Anatomical and Molecular Pathology, Faculty of Medicine, University of Coimbra, 3000-548 Coimbra, Portugal; 7 Allergy and Clinical Immunology Unit, Coimbra Hospital and University Center, Unidade Local de Saúde de Coimbra, 3004-561 Coimbra, Portugal; 8 Institute of Immunology, Faculty of Medicine, University of Coimbra, 3000-548 Coimbra, Portugal; 9 Laboratory of Biostatistics and Medical Informatics (LBIM), Faculty of Medicine, University of Coimbra, 3000-548 Coimbra, Portugal; 10 Centre for Innovative Biomedicine and Biotechnology (CIBB), University of Coimbra, 3000-504 Coimbra, Portugal

**Keywords:** Luminal A breast cancer, sentinel lymph node, immune microenvironment, cancer genomics, OSNA

## Abstract

**Simple Summary:**

In this study, the researchers aimed to improve the prognostic information provided by the standard One-Step Nucleic Acid Amplification (OSNA) assay used to detect breast cancer (BC) metastasis in sentinel lymph nodes (SLNs). They analysed the expression of an immune gene panel in SLNs from Luminal A early-stage BC (cT1-T2 N0) patients, including those with and without subclinical metastasis. The study identified two distinct immune response profiles—one showing an adaptive anti-tumour immune response, and another with a more undifferentiated response. The researchers also identified seven key immunoregulatory genes that could serve as potential targets for immunotherapy. These findings suggest that analysing immune gene expression in SLNs can provide additional prognostic information beyond the OSNA assay results and may help guide personalised treatment approaches for early-stage BC patients.

**Abstract:**

In the management of early-stage breast cancer (BC), lymph nodes (LNs) are typically characterised using the One-Step Nucleic Acid Amplification (OSNA) assay, a standard procedure for assessing subclinical metastasis in sentinel LNs (SLNs). The pivotal role of LNs in coordinating the immune response against BC is often overlooked. Our aim was to improve prognostic information provided by the OSNA assay and explore immune-related gene signatures in SLNs. The expression of an immune gene panel was analysed in SLNs from 32 patients with Luminal A early-stage BC (cT1-T2 N0). Using an unsupervised approach based on these expression values, this study identified two clusters, regardless of the SLN invasion: one evidencing an adaptive anti-tumoral immune response, characterised by an increase in naive B cells, follicular T helper cells, and activated NK cells; and another with a more undifferentiated response, with an increase in the activated-to-resting dendritic cells (DCs) ratio. Through a protein—protein interaction (PPI) network, we identified seven immunoregulatory hub genes: *CD80*, *CD40*, *TNF*, *FCGR3A*, *CD163*, *FCGR3B*, and *CCR2*. This study shows that, in Luminal A early-stage BC, SLNs gene expression studies enable the identification of distinct immune profiles that may influence prognosis stratification and highlight key genes that could serve as potential targets for immunotherapy.

## 1. Introduction

In 2020, breast cancer (BC) became the most diagnosed cancer worldwide, representing the leading cause of cancer-related mortality among women [1]. The presence of axillary lymph node (LN) metastasis and the immune response to cancer have a determinant role in disease progression [2,3]. From the early stages of the disease, an intense and dynamic interplay is established between tumours and LNs [4]. The axillary sentinel LN (SLN) is the first tumour-draining LN, making it the ideal site to explore the immune response to cancer. Even before SLN invasion, soluble factors derived from tumour cells reach the SLNs. These factors may establish favourable microenvironments for successful colonisation (pre-metastatic niche) or, instead, initiate a defensive immune response [4,5]. Studies of SLN in node-negative BC patients, and comparisons with LNs from women without cancer, confirm the SLN’s ability to recognise the tumour before the presence of metastasis [6,7]. If a productive immune recognition of cancer cells is established at the primary site, antigen-presenting cells (APCs), such as DCs, will be activated and migrate to LNs where they present tumour antigens to T cells [5]. Upon activation, cytotoxic T lymphocytes and CD4^+^ T cells return to the tumour and specifically recognise and eliminate cancer cells. In fact, tumour-infiltrating leukocytes in LN-positive patients show higher tumour destruction activity than those from LN-negative patients [8]. In this sense, LNs are believed to be the main hubs responsible for orchestrating an adaptive immune response against BC [5]. The presence of tumour cells in the SLN appears to be the seed for distant metastasis and is recognised as a major prognostic factor associated with cancer progression and poor patient outcome [5].

Many studies designed to unravel the immune response involved in locoregional metastasis describe a progressive state of immunosuppression from non-invaded to invaded LNs, as compared to healthy, non-tumour-draining LNs. This immunosuppressive state includes increased levels of exhausted CD4^+^ and CD8^+^ T cells expressing CTLA-4 and PD-1 [7] or TIGIT [9] immune checkpoints proteins, and increased frequencies of activated regulatory T cells (Tregs) [10] and myeloid-derived suppressor cells (MDSCs) [7]. Additionally, there is evidence of a decreased frequency of CD163^+^ macrophages in the SLN of patients with BC compared to non-tumour-draining LNs [11], particularly for invaded SLNs. In vitro stimulation studies of immune cells isolated from LNs showed that in tumour-draining LNs, the production of cytokines was reduced, particularly in positive SLN, when compared to normal LN. This included diminished levels of IL-8, IL-1β, IL-6, TNF-alpha, and IL-10 in DC, as well as reduced interferon gamma to IL-4 ratio in T cells [7]. However, other studies describe a more complex scenario. In a comparison between SLNs and other axillary non-SLNs from LN-negative patients, Matsuura et al. (2006) found evidence of impairment of DC maturation and consequent compromised differentiation of T helper cells in SLNs [6]. Conversely, SLNs from node-positive patients showed markers of DC maturation and upregulation of T helper 1 (Th-1) response [6]. Additionally, Rye et al. (2022) when characterising LNs immune cell subpopulations using single-cell studies and CyTOF, found evidence of an immune suppression response only in non-SLN-invaded axillary LNs [9]. No significant difference was observed between SLN-negative and SLN-positive samples, except for a subtle increase in a small population of transient, unprimed, dual-labelled CD4 T cells (CD45RA^+^CD45RO^+^) in positive SLNs [9]. Also, in vitro-stimulated T cells were described to release similar cytokine levels (IL-4, IL-6, IL-10, TNF-alpha and IL-17A) in non-invaded BC SLN and normal LN [7]. Regarding natural killer (NK) cells, no significant differences in LN infiltration have been found between invaded and non-invaded tumour-draining LN or even when compared with normal LNs [12]. However, in BC LNs, NK cells exhibited increased expression of activating receptors, such as NKG2D and DNAM-1, as well as the inhibitory receptor NKG2A [12]. In multiple studies, invaded and non-invaded SLNs exhibit phenotypic resemblance, with statistically significant differences observed only for a limited set of parameters, including cytokines and immune cell subsets [7,9,10,12]. Differences are mostly observed when comparing invaded LNs with normal LNs [7,11]. Another relevant point across all studies is the high intragroup heterogeneity of the results [6,7,9,11,12,13,14,15]. Evidence suggests that SLN response may serve as an indicator of the systemic immune response. In a comparison of BC patients with or without axillary metastasis, Zuckerman et al. (2013) found that in the group with axillary metastasis, immune cells isolated from both invaded and non-invaded LNs, the primary tumour, as well as from peripheral blood, showed similar gene expression profiles. These profiles were characterised by the downregulation of genes associated with immune-related pathways and the upregulation of genes associated with tumour-promoting pathways [16]. Moreover, the type of response of LNs to the primary tumour and to metastasis may predict disease evolution. Longer disease-free survival (DFS) was associated with an increase in T and B cells in SLNs in different BC subtypes, regardless of the nodal status [13].

In our previous study [17], we analysed the expression of immune-related genes in SLNs from Luminal A early-stage BC (cT1-T2 N0) patients [18]. SLN samples were obtained from the discarded homogenised lysates of the One-Step Nucleic Acid Amplification (OSNA) assay, a standard procedure for assessing SLN subclinical metastasis [19]. This assay uses reverse transcription loop-mediated isothermal amplification (RT-LAMP) to detect cytokeratin 19 (CK19) mRNA in the lysed SLNs [19]. The cut-off levels determined by Tsujimoto et al. distinguish between the presence of macrometastases, micrometastases, and SLNs that are either non-invaded or contain isolated tumour cells [19]. When non-invaded SLNs were compared to SLNs with macrometastasis, 11 differentially expressed genes (DEGs) were identified, all of which were upregulated in SLNs with macrometastasis. These genes are mainly expressed by cancer cells and encode proteins involved in cancer aggressiveness with an impact on the immune response. Nevertheless, hierarchical clustering based on these DEGs did not completely align with the SLN OSNA classification, suggesting the interference of other factors besides the presence of metastasis. In this study, we used an unsupervised approach to explore immune-related profiles in SLNs, aiming to enhance our understanding of SLN immune response, identify profiles that may impact prognosis stratification, and highlight potential targets for immunotherapy.

## 2. Materials and Methods

### 2.1. Data Acquisition

This study analyses RNA-Seq data obtained from discarded OSNA SLNs lysates of 32 women with Luminal A early-stage BC (cT1-T2 N0). The complete methodology for patient recruitment, along with the inclusion and exclusion criteria and SLN biopsy, is detailed in Gante et al. (2022) [17]. Tumours were classified as Luminal A according to international guidelines (Estrogen Receptors-positive, HER2-negative, Ki67 < 20% and Progesterone Receptors ≥ 20% [17,18]. The samples included 16 SLNs without metastasis (pN0), 7 SLNs with micrometastasis (pN1mi), and 9 SLNs with macrometastasis (pN1) [17]. All the samples had been previously sequenced using the Oncomine™ Immune Response Research Assay (Thermo Fisher Scientific, Carlsbad, CA, USA) panel [17], which assesses the expression levels of 395 immunoinflammatory-related genes. Targeted RNA-Seq data are available in NCBI’s Gene Expression Omnibus database (accession number GSE210006).

### 2.2. K-Means Clustering Analysis

Aiming to group SLN samples that exhibited similar expression patterns, the K-Means algorithm was implemented with the K-Means function from the Stats R Package (version 4.2.2) on gene expression data normalised by the DESeq2 R package (version 1.36.0) [20]. Briefly, the DESeq2 normalisation involves calculating the ratio of each read count to the geometric mean of read counts for that gene across all samples [20]. After normalisation, the data were filtered to include only those genes with a variance and mean greater than 50. The maximum number of iterations was set to 1000. The Factoextra R package (v1.0.7) was used to determine the optimal number of clusters (k) using the average silhouette method and to visualise the results of the K-Means clustering. We performed a Chi-Square analysis to assess whether there was a statistically significant (*p* < 0.05) association between the distribution of SLN samples across the clusters and their respective classifications relative to the presence (pN1mi + pN1) or absence (pN0) of metastasis.

### 2.3. Identification of Differentially Expressed Genes (DEGs)

Using DESeq2 (version 1.36.0) [20] and a false discovery rate (FDR) threshold of 0.05 to correct for multiple comparison errors (Benjamini and Hochberg method), DEGs were identified from the comparison of the two clusters (C1 and C2) previously obtained using the K-Means analysis. A log2 fold change cut-off was set to filter DEGs for changes greater than the absolute value of 0.58 (corresponding to a fold change > 1.5 or <0.67). Plots were generated using the ggplot2 package in R (version 3.4.1).

### 2.4. Gene Set Enrichment Analysis (GSEA)

To identify gene-signature-based differences between the C1 and C2 SNL samples, GSEA was performed using immune-related gene signatures. The GSEA function from the clusterProfiler R package (version 4.12.0) [21] was used to analyse the list of all genes used for differential expression analysis. The genes were ranked by their log2 fold changes, and the GSEA was performed using the default parameters, including the fgsea method with 1000 permutations and the criteria for selecting gene sets with a minimum of 10 genes and a maximum of 500 genes. An FDR of <0.05, calculated using the Benjamini–Hochberg adjustment method, was considered statistically significant. The gene signatures used in this study were compiled from several sources. Three collections from the Molecular Signatures Database (MSigDB) [22] were included: Curated gene sets (C2), Hallmark gene sets (H), and Immunologic signature gene sets (C7). Additionally, two libraries were retrieved from the Enrichr gene set search engine [23]: a filtered library of 100 gene sets related to the term “lymph node” and Tabula Sapiens. Furthermore, two sets of gene signatures previously used to annotate immune cells in a tumour context were also included: 22 signatures from Azizi et al. (2018) [24] and 29 signatures from Fan et al. (2021) [25].

### 2.5. Protein–Protein Interaction (PPI) Network and Hub Gene Identification

The online tool STRING (version 12) [26] was used to construct a PPI network to obtain potential interactions among proteins encoded by the DEGs identified in the study. A confidence score cut-off of >0.7 was set to ensure high-confidence interactions. The resulting network was then loaded into CytoScape software (version 3.10.2) [27] for further analysis. To identify the most influential nodes in the network, the CytoHubba plug-in [28] was used to score the nodes based on various network features. Six topological analysis methods were employed: Closeness, Degree, Edge Percolated Component (EPC), Maximal Clique Centrality (MCC), Maximum Neighborhood Component (MNC), and Radiality [28], based on the recommendations made by Wang et al. (2022) [29]. The scores from each method were then ranked and the top 10 DEGs were selected [28]. The hub genes were identified by overlapping the top DEGs of each scoring method. 

### 2.6. Data Integration of Transcriptome Profiles of Normal LN (NLN)

Gene expression data from five normal LN (NLN) samples, generated using the Illumina platform and normalised by FPKM (fragments per kilobase of exon model per million mapped reads), were retrieved from the ArrayExpress database (accession number E-MTAB-1733). Only genes included in the Oncomine™ Immune Response Research Assay panel (Thermo Fisher Scientific, Carlsbad, CA, USA) were analysed.

### 2.7. Comparative Analysis of Immune Gene Expression between SLNS and NLNs

Four groups were analysed: the C1 cluster, C2 cluster, OSNA pN0 SLNs, and NLNs. To account for platform-specific and normalisation-related differences between the datasets (SLNs and NLNs), a rank-based approach was implemented, enabling the evaluation of relative expression levels of genes. First, genes were ranked within each patient according to their expression values. Then, each expression value was replaced by its ranking position value. Finally, an independent t-test was used to compare the ranking position values between the different groups. The FDR threshold of 0.05 was applied to correct for multiple hypothesis testing (Benjamini and Hochberg method). A statistical analysis was conducted using Microsoft Excel and the R package stats (version 4.2.2). Plots were generated using the ggplot2 package in R (version 3.3.6).

### 2.8. In Silico Quantification of Immune Cell Populations

CIBERSORTx [30] was used to estimate the proportions of immune cell types in SLNs using gene expression data. For this analysis, we selected the LM22 microarray-derived signature matrix, which consists of 547 genes distinguishing 22 human hematopoietic populations, including T cell types, B cells (naive and memory), plasma cells, NK cells, and myeloid subsets [31,32,33]. The number of permutations was set to 1000. As LM22 was derived from microarray data, and the OSNA SLN mixtures were derived from RNA-Seq profiles, the batch-correction option was used to minimise the impact of cross-platform variation on the results. The absolute mode reported the results as relative fractions normalised to 1 for all cell subsets. CIBERSORTx *p*-value, reflecting the statistical significance of the deconvolution results, was used to filter out results with less significant fitting accuracy.

### 2.9. Statistical Analysis Comparing Immune Cell Populations in SLN Clusters

The normal distribution of cell population scores was evaluated through the Shapiro–Wilk test. The F-test was used to assess whether the variances of the two clusters were similar. Mean values and standard deviations for each cell subset were calculated for each cluster. A comparison of the cell population scores was performed using the appropriate statistical tests (Supplementary Table S1), and the statistical significance was set at *p*-value < 0.05. A statistical analysis was performed with the R package stats (version 4.2.2). Plots were generated using the ggplot2 package in R (version 3.3.6).

## 3. Results

### 3.1. SNL Samples Segregate into Two Clusters Regardless of the Presence of Metastasis

**Figure 1:**
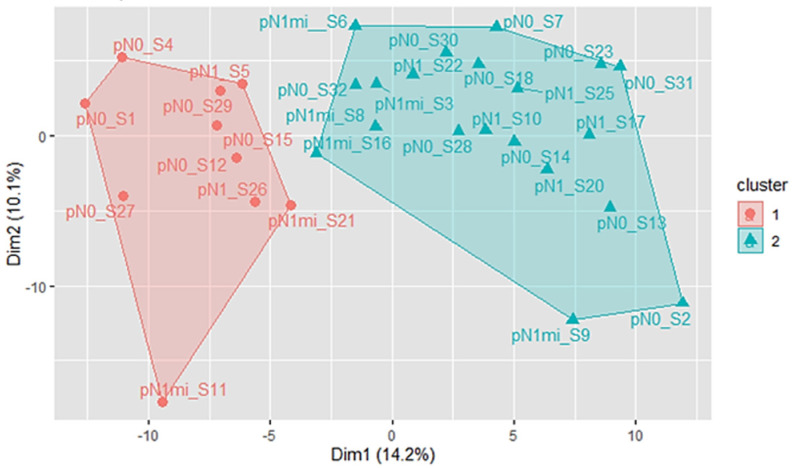
K-means clustering results when k = 2, after excluding the outlier samples S19 and S24. PCA algorithm was used to reduce the dimensionality of the data. The first two principal dimensions are used for visualisation. C1 SLN samples are indicated by red dots, while C2 SLN samples are indicated by green triangles. SLNs without metastasis are represented by the prefix pN0 followed by the sample ID, SLNs with micrometastasis are represented with the prefix pN1mi followed by the sample ID, and the SLNs with macrometastasis are represented with the prefix pN1 followed by the sample ID.

The unsupervised analysis identified two distinct clusters of samples (Figure 1, Table S1). These clusters maximise the average silhouette width values (Figure S1a), indicating a strong and well-defined separation between the clusters. Ten samples were assigned to cluster 1 (C1) and 20 to cluster 2 (C2). Two outlier samples, S19 and S24, were excluded from the analysis (Figure S1b). There was no significant association between the clusters C1 and C2, and the OSNA classification based on the presence of metastasis (pN0: 60% in C1 vs. 50% in C2; pN1mi: 20% in C1 vs. 25% in C2; pN1: 20% in C1 vs. 25% in C2; χ^2^(2) = 0.268, *p* = 0.875, N = 30). Other parameters such as tumour-infiltrating lymphocytes (TILs) and the total number of invaded LNs were also similar between the two clusters.

### 3.2. SNL Clusters Exhibit Distinct Immune System Expression Profiles

**Figure 2:**
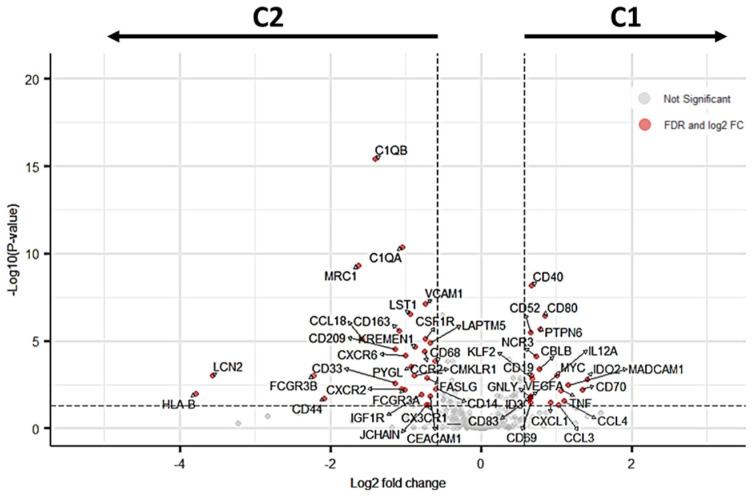
The volcano plot displays the relationship between the magnitude of gene expression change (log2 of fold change on the *x*-axis) and the statistical significance of this change (−log10 of false discovery rate (FDR) on the *y*-axis). Red points represent significant DEGs identified when comparing C1 vs C2 using an FDR < 0.05 (horizontal dashed line) after post-hoc filtering performed on |log2(FC)| > 0.58 (vertical dashed lines). DEGs downregulated in C1 correspond to DEGs upregulated in C2.

A total of 51 DEGs were identified when comparing C1 vs C2 (FDR < 0.05). Twenty-two of the 395 analysed genes were upregulated (5.5%), whereas 29 (7.3%) were downregulated in C1 compared to C2 (Figure 2; Supplementary Tables S2 and S3). Subsequent analyses will consider that DEGs downregulated in C1 correspond to DEGs upregulated in C2.

**Figure 3:**
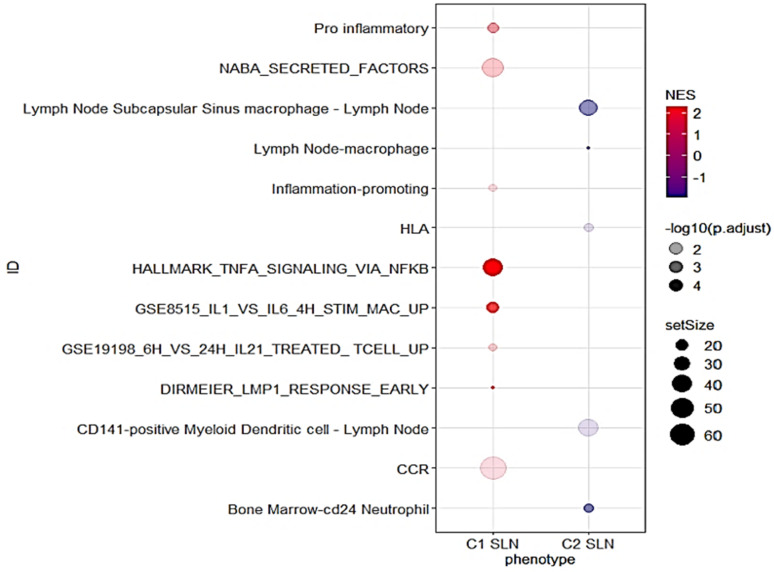
Dot plot for selected GSEA results. The *x*-axis represents the different SLN clusters (C1 and C2). The *y*-axis shows the ID of the gene sets. The size of the dots represents the gene set size, the colour represents the Normalized Enrichment Score (NES), and the transparency represents the negative logarithm of the adjusted *p*-value (−log10(p.adjust)).

To further explore the differences in gene signature enrichment between the C1 and C2 SLNs clusters, a GSEA analysis was conducted using immune-related gene signatures. The results showed that gene signatures enriched in C1 SLNs were mainly associated with NF-kappa B activation via TNF-alpha signalling, activation and proliferation of B cells (DIRMEIER_LMP1_RESPONSE_EARLY), secreted soluble factors of the matrisome (NABA_SECRETED_FACTORS), the human stimulated macrophage transcription profile (GSE8515_IL1_VS_IL6_4H_STIM_MAC_UP), T cell genes upregulated by IL21 (GSE19198_6H_VS_24H_IL21_TREATED_TCELL_UP), pro-inflammatory response inflammation-promoting genes, and chemokine receptor gene signature (CCR) (Figure 3, Supplementary Table S4). In contrast, the majority of the gene signatures enriched in C2 SLNs were related to the innate immune response, including lymph node subcapsular sinus macrophages and lymph node CD141-positive Myeloid Dendritic cells (Figure 3, Supplementary Table S4).

### 3.3. Hub Genes Associated with SLN Clusters

**Figure 4:**
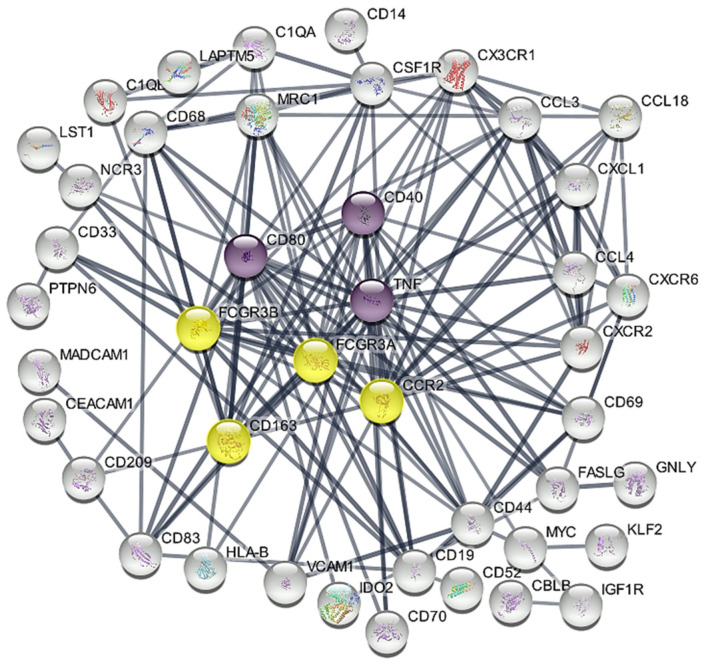
STRING PPI network based on the identified 51 immune-related DEGs. Upregulated hub genes in C1 are represented by purple circles, while upregulated hub genes in C2 are marked by yellow circles. The remaining DEGs are shown in white circles. Disconnected nodes in the network have been hidden.

The network contained 51 nodes and 153 edges (expected number of edges: 119) (Figure 4) (PPI enrichment *p*-value: 1.53 × 10^−3^). Using the CytoHubba plug-in and analysing the gene network with six topological analysis methods, we identified seven hub genes that consistently appeared among the overlapping top 10 genes: *CD80*, *CD40* and *TNF* (upregulated in C1) and CD163, FCGR3A, *FCGR3B* and *CCR2* (upregulated in C2) (Figure S2).

### 3.4. Comparative Gene Expression Analysis of SLNs and NLN

**Figure 5:**
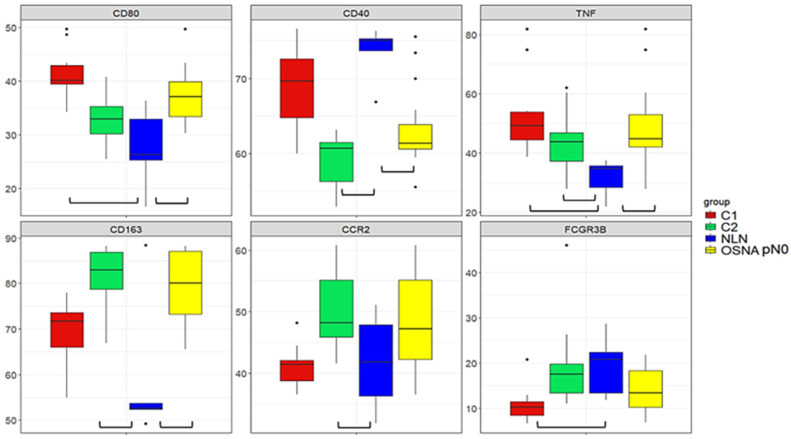
Boxplots for hub genes in C1, C2, NLNs and OSNA pN0 SLNs groups. The *x*-axis refers to the different groups being compared, and the *y*-axis represents the ranks assigned to each gene within each group. Only genes that show a significant difference (FDR < 0.05) in expression when comparing the NLN group to the other groups (OSNA pN0, C1, or C2) are shown.

In the normal LN (NLN) dataset, we found 380 out of 395 genes from the Oncomine™ Immune Response Research Assay panel. These genes included 47 out of the 51 DEGs identified. All 380 genes were used in subsequent analyses. We compared the relative expression of the hub genes between healthy NLNs, SLNs without metastasis (OSNA pN0), and C1 and C2 clusters using a rank-based approach. Our findings show that NLN and OSNA pN0 SLNs have a different profile for the majority of the hub genes (Figure 5; Table S5), the relative levels of expression being higher for *CD80*, *TNF*, and *CD163* and lower for *CD40* in OSNA pN0 SLNs. Comparing the NLN group with C1 and C2, *TNF* exhibits significantly higher relative expression in both clusters. The same trend is observed for *CD80* in C1 and for *CD163* and *CCR2* in C2. On the other hand, *CD40* shows significantly lower relative expression in C2 compared with the NLN group and *FCGR3B* is significantly lower in C1. Regarding *FCGR3A*, the levels of expression were similar between the four groups. The overall comparison of the relative expressions of the 47 DEGs between C1 and the NLN group reveals statistically significant differences in 48.9% of these genes (Table S6), with the relative expression values corrected for multiple comparison error. This percentage increases to 63.9% when comparing C2 with the NLN group. Finally, comparing the relative expression of 376 immune-related genes between OSNA pN0 SLNs and the NLN group reveals statistically significant differences in 64.6% of the genes (Table S7). The analysis comprised 376 out of the 380 genes shared by the two data sets, as four non-DEGs were excluded. This exclusion was due to their standard deviation being equal to zero, resulting from identical rank values across different groups. Since *CD80* expression from APCs, such as DCs, B cells and macrophages, plays a crucial role in providing essential co-stimulatory or inhibitory signals to T cells through their T-cell receptors CD28 and CTLA-4, respectively [34], we evaluated the ratio between the normalised expression of their levels. The results showed that the average expression level of *CD28* was seven times higher than *CTLA-4* in C1, C2, and in OSNA pN0 samples (Table S17). In contrast, for the NLN samples, the ratio was 2.4 (Table S8).

### 3.5. Comparison of Immune Cell Populations between SLN Clusters

**Figure 6:**
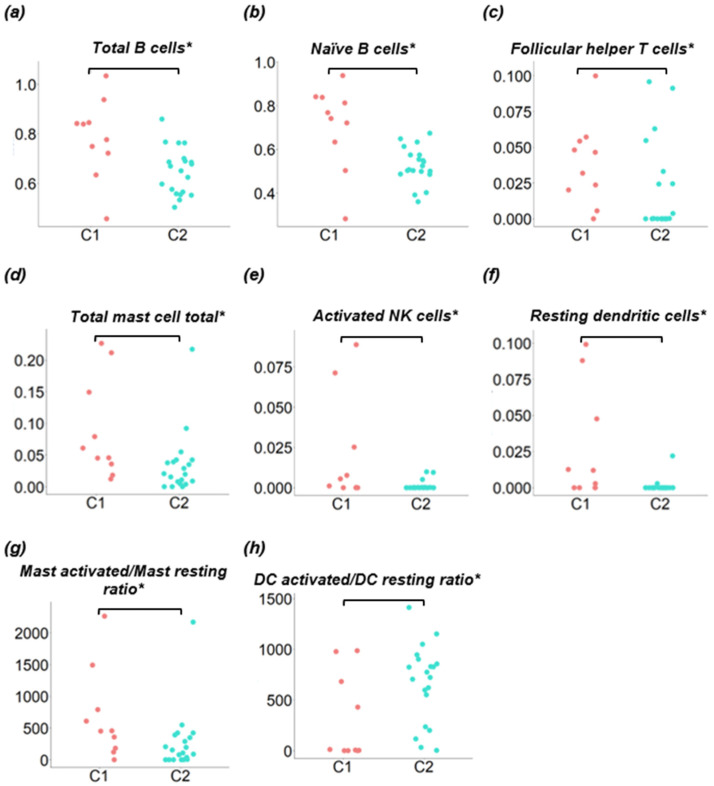
Comparison of immune cell populations between clusters C1 and C2. CIBERSORTx immune cell fractions were determined for each sample; each dot represents one sample. The *x*-axis refers to the clusters being compared (C1 and C2) and the *y*-axis represents the CIBERSORTx cell population scores. * *p* < 0.05.

One downside of the OSNA assay is the destruction of the SLN samples, preventing further structural studies that could provide useful information on immune response. To overcome this limitation, we used CIBERSORTx to estimate the immune cell populations present in the SLNs homogenised samples based on gene expression data (Table S9). All the samples showed a deconvolution *p*-value < 0.05 and were included for further analysis. Cluster C1 was characterised by a statistically significant increase in the populations of both acquired and innate immune response, namely, total B cells, naive B cells, follicular T helper cells, total mast cells, activated NK cells, resting dendritic cells (DC), and activated-to-resting mast cells ratio (Figure 6a–g; Table S10). Cluster C2 was characterised by very low levels of follicular T helper cells, activated NK cells and resting DC. Only the activated-to-resting DC ratio was increased in C2 (Figure 6h). None of the macrophage subpopulation types or M2/M1 ratio showed to be significantly enriched in any of the clusters (Tables S9 and S10). Other ratios such as the neutrophil-to-lymphocyte ratio (NLR) and the monocyte-to-lymphocyte ratio (MLR) did not show statistically significant differences between the clusters (Table S9).

## 4. Discussion

While the immune system’s response to BC is recognised as crucial for disease progression, the current risk stratification of patients is based only on tumour biology and disease staging. Our aim was to analyse the immune-related gene expression profile of SLNs to identify potential predictive and prognostic markers. We specifically analysed Luminal A early-stage BC (cT1-T2 N0) as there is a lack of studies investigating the immune profiles of the LN microenvironment in these tumours. Although Luminal A BC generally exhibits a favourable prognosis, its high prevalence raises concerns, as some of these patients may still develop metastasis.

The unsupervised analysis of immune gene-related expression data grouped the SNL samples into two clusters. These clusters, C1 and C2, were similar for the distribution of non-invaded (pN0), micro (pN1mi) and macrometastatic (pN1) LNs, for the total number of invaded LNs, as well as for TILs in the primary tumour. It is important to consider that SLNs classified as non-invaded (pN0) may include SLNs that have successfully eliminated cancer cells or harbour isolated cancer cells below the OSNA assay detection limit. Additionally, the expression of immune-related genes in pN0 SLNs may be triggered by soluble mediators and cancer cells’ neoantigens in APCs drained from the primary tumour [4,5]. Other authors have compared the immune profiles of invaded and non-invaded sentinel and non-sentinel lymph nodes. These studies describe a complex immune profile that is not always directly correlated with the presence of metastasis [7,9,13]. The differential expression analysis between the two clusters revealed fifty-one DEGs (12.9% of the analysed genes): twenty-two were upregulated and 29 were downregulated in C1 compared to C2. As none of these DEGs were identified in our previous study [17] comparing invaded and non-invaded SLNs, the immune cell population is expected to be the major source of their expression. Some of the DEGs have immunomodulatory roles, such as *IDO2* and *CD70*, upregulated in C1, and *CD163* and *CD44* upregulated in C2. Aberrant expression of corresponding proteins has been described in some types of cancer cells and associated with worse prognosis, being currently explored as therapeutic targets [35,36]. However, the meaning of their expression in tumour-draining LNs immune cells’ remains to be determined. GSEA analysis suggested that the two clusters exhibit different immunological profiles. Cluster C1 revealed a more diverse profile, with enrichment in gene signatures related to the activation of the innate and the adaptive immune responses. On the other hand, C2 enrichment analysis was almost exclusively related to the innate immune system, specifically referring to lymph node subcapsular sinus macrophages and lymph node CD141-positive myeloid DCs. Using the 51 DEGs, a protein—protein interaction (PPI) network allowed the identification of seven hub genes candidates for prognosis and predictive markers: *CD80*, *CD40* and *TNF* upregulated in C1, and *CD163*, *FCGR3A*, *FCGR3B* and *CCR2* upregulated in C2. *CD80* is a membrane protein that belongs to the B7 family of co-stimulatory molecules and is primarily expressed in APCs, although it can also be expressed by other immune cells [37]. The binding of CD80 to CD28, in combination with the T-cell receptor (TCR) signal, provides a co-stimulatory signal necessary for complete T-cell activation and initiation of adaptive immunity [34]. On the other hand, CTLA-4 competes with CD28 to bind CD80 with higher affinity, downregulating the activation process [34]. When assessing specific immunological differences between SLNs from BC patients and NLNs from healthy individuals, we found that the *CD28*/*CTLA-4* ratio was much higher in the C1 and C2 clusters and in the OSNA pN0 samples than in the NLNs, suggesting an immune response activation in tumour-draining SLNs, particularly in C1 SLNs, which exhibited higher *CD80* expression levels. CD40 is another co-stimulatory receptor molecule that is mainly expressed in APCs, such as B cells, macrophages, and DCs, being required for their activation. Its ligand, CD40L, is expressed primarily by activated T cells [38,39]. The CD40-CD40L interaction plays a crucial role in regulating the immune response: it promotes CD80 expression [38,39], secretion of TNF-alpha, activates several signal transduction pathways, including the NF-kappa B [38] and induces differentiation of activated B cells into memory B cells and formation of germinal centres (GCs) [39]. Therefore, the differential expression of *CD40* may play a key role in the C1 profile. Interestingly, LNs germinal centres were associated with a lower risk of developing distant metastasis in triple negative BC patients with involved LNs [40]. Also, vaccination studies using CD40-activated B cells acting as APCs could activate and expand anti-tumour naive and memory T cells [41]. *TNF*, also identified as the C1 hub gene, encodes tumour necrosis factor (TNA-alpha), a cytokine with pleiotropic effects mainly secreted by activated macrophages, T and B lymphocytes, and NK cells [42]. TNF-alpha and CD40 are major inducers of NF-kappa B signalling, a key regulator of immune responses: TNF-alpha stimulates the canonical pathway, which is triggered mainly by external stimuli and activates either the innate or the adaptive responses, whereas CD40 activates the non-canonical pathway, characterised by a slow and more persistent and specific response, and with an important role in the development of LNs germinal centres (review in Yu et al., 2020 [43]). Canonical NF-kappa B signalling inhibition has been proposed for cancer therapy and further research should analyse TNF-alpha and CD40 roles as SLNs predictive markers. Considering the C2 upregulated hub genes, *CD163* encodes a scavenger receptor that binds circulating hemoglobin-haptoglobin complexes and mediates anti-inflammatory cellular functions [44]. Despite being considered a useful marker for discriminating M2 from M1 macrophages, CD163 can also be expressed in monocytes and myeloid DCs [45]. In LNs, CD163+ macrophages were mainly found in the perifollicular and medullary areas [11]. Although a high density of CD163+ tumour-associated macrophages (TAMs) in BC is associated with poor survival outcomes [46], Mansfield et al. (2012) found that in BC SLNs, the presence of sinusoidal CD163+ macrophages was associated with favourable nodal status, suggesting that the impact of CD163+ macrophages may depend on the microenvironment [11]. CD163 expression is induced by anti-inflammatory stimuli like IL-10 and suppressed by pro-inflammatory mediators like TNF-alpha [44], which is downregulated in C2 SLNs. When studying macrophage subpopulations in BC-tumour-draining LNs using a scRNA-seq approach, Xu et al. (2021) found a polarisation towards M2 subtypes characterised by expression of *CD163*, *CCL18*, *CCL13*, and *MRC1* [47]. M2 markers, *CCL18*, *MRC1*, and *CD163* are C2 DEGs. However, since in this study we quantified *CD163* mRNA in the hole homogenised SLN, it is not possible to specify the cell or cells of origin. *FCGR3A* and *FCGR3B*, which were also identified as C2 hub genes, are members of the Fc receptor with low affinity for the IgG (FCGR3) family, and mediate antibody-dependent cell-mediated cytotoxicity by macrophages and NK cells [48,49]. *FCGR3A* encodes the transmembrane Fc gamma IIIa receptor which is expressed in most effector cells such as macrophages, NK, and in gamma delta T cells [50]. In contrast, *FCGR3B* encodes a glycosylphosphatidylinositol (GPI)-anchored protein that is expressed constitutively by neutrophils, and after gamma-interferon stimulation, by eosinophils [50]. The role of *FCGR3A* in cancer has recently gained significant interest, with studies proposing it as a poor prognostic biomarker in prostate cancer and lower-grade glioma [48,51]. However, the expression of both genes within tumour-draining LNs remains unexplored. Lastly, the C-C motif chemokine receptor 2 gene (*CCR2*) encodes the transmembrane receptor for monocyte chemoattractant protein-1, CCL2, a chemokine that mediates monocyte chemotaxis. CCR2 is primarily expressed on the surface of immature DCs, basophils, NK cells, and T lymphocytes [52] whereas CCL2 is expressed by many immune and non-immune cells. Activation of CCL2/CCR2 axis has been described to promote an immune suppressive tumour microenvironment by attracting suppressive monocytes and Tregs, thus favouring tumour progression and metastasis (review in [53,54]). Strategies to target CCL2/CCR2 axis as cancer therapy are under investigation.

To gain further insight into the SLN immune response to the tumour, we compared the expression of the hub genes between C1, C2, SLNs with no metastasis (OSNA pN0) and normal, non-tumour-draining LNs (NLNs). The major finding was that except for *FCGR3A*, for which there was no significant variation of the expression levels between the different groups, non-metastatic SLNs had a different profile from NLNs, confirming the immune recognition of the primary tumour.

The quantification of the immune cell composition of the SLNs also suggests important differences between the two clusters, although great heterogeneity was observed within each cluster. C1 was characterised by a statistically significant increase in almost all immune cell populations, more evident for naive B cells, follicular T helper cells (Tfh), and activated NK cells. Tfh cells are essential to LNs germinal centre formation, where they promote the clonal expansion and selection of B cells via CD40-CD40L, a C1-associated hub gene [55]. C2 showed reduced levels of almost all immune cell populations, and only the activated-to-resting DC ratio was increased in this cluster. Although NK activated cells were barely represented in both clusters, their levels were higher in C1, and only three samples in C2 showed residual values. These cells, part of the innate immune system, can directly induce the death of tumour cells. Additionally, their cytokine production influences T and B cells. Although mRNA of *CD163* hub gene was upregulated in C2, no statistically significant differences were observed in the inferred abundance of macrophages between the two clusters. However, as previously stated, we did not quantify the membrane protein, but mRNA, and cell population inference is based on a score of multiple transcripts with cell-specific differential expression.

The overall results suggest two distinct immune profiles for SLNs, independent of the presence of metastasis: one associated with C1, characterised by preponderant adaptive anti-tumoural immune response, and another associated with C2, exhibiting a more undifferentiated response. C2 may correspond to an early stage, delayed, immune response or to an immune suppressive response. The two profiles may be related to interindividual variability of immune response or with specific tumour characteristics and may impact prognosis. Besides the need for long-term, prospective studies with a larger sample, single-cell sequencing and spatial transcriptomics would help elucidate the predictive role of the immune response of SLNs in BC. Currently, a limited number of studies in BC have applied single-cell sequencing to investigate differences in immune cell populations within tumour-draining LNs [24,47]. However, the reduced number of patients requires additional validation studies.

This is an exploratory study. Validation of results using independent and larger datasets, along with prospective studies would verify the link between the identified immune profiles, hub genes expression and patient prognosis. However, for this specific group of patients, prospective studies would require long follow-up periods. The use of a specific gene panel, like the one used in this study, limits the identification of biomarkers and target genes but offers the advantage of more optimised results. The inclusion of normal, non-tumour-draining LN (NLN) in high-throughput gene expression studies is rare due to its limited availability and high cost. Therefore, to compare our results with NLNs, we used data from a public database. As no platform-compatible datasets were available, we opted to implement a rank-based approach. Using ranks, the analysis was based on the relative ordering of gene expression levels rather than the absolute expression values themselves. This approach minimises the influence of platform-specific and normalisation-related differences on the results by ensuring that the relative ordering of genes remains consistent across samples. The observed high interindividual variability in the immune response within each cluster is expectable and described by other authors using either gene expression assays or protein-based approaches [6,7,9,11,12,13,14,15]. Although the use of OSNA remaining lysate requires the application of bulk RNA sequencing, it is currently the only material available to study SLNs of early-stage BC patients. Despite these limitations, using SLNs to characterise the immune response to the tumour rather than directly exploring the tumour microenvironment offers several advantages: beyond the key role of LNs in orchestrating the immune response against cancer, they provide abundant and accessible material for molecular studies, particularly those processed by the OSNA assay, overcoming the current limitation of the reduced size of primary tumours. Finally, immune characterisation of SLNs may also help overcome a major drawback of the OSNA assay: its inability to explore other prognosis and predictive markers only revealed by SLN architecture, as the SLN is destroyed during the procedure [17].

## 5. Conclusions

In the era of precision medicine, this study strengthens the importance of exploring BC SLN immune response as a source of predictive markers. Two distinct immune profiles within the SLNs of Luminal A early-stage BC patients, independent of the presence of metastasis, were identified: one was characterised by an adaptive, anti-tumoural immune response, whereas the other exhibited a more undifferentiated state suggesting immune suppression. The fact that these immune profiles are independent of the presence of subclinical metastasis suggests that patients with the same OSNA result may have different prognoses and could benefit from tailored treatment approaches. Improved patient stratification would be important for customising local management strategies, such as axillary dissection and radiotherapy, as well as optimising systemic therapy. It would also help in proposing stricter follow-up protocols for higher-risk patients. Hub genes, which encode immunomodulatory molecules that are potential therapy targets, were also identified for each SLN immune profile. Both the immune profiles and hub genes should be explored as predictive markers for immunotherapy. The microenvironment of SLNs in other BC intrinsic subtypes may exhibit different profiles. Prospective validation studies with larger population samples are currently underway.

## Data Availability

The RNA-Seq and SLN OSNA classification data presented in this study data are available in NCBI’s Gene Expression Omnibus database (accession number GSE210006).

## Supplementary Materials

The following supporting information can be downloaded at: https://www.mdpi.com/article/10.3390/cancers16162881/s1, Figure S1. (a) Visual assessment of the K-means clustering results when K = 4. (b) Average silhouette method for different values of k computed after excluding the outlier samples S19 and S24. Figure S2. Hub genes were identified by overlapping the top DEGs of each scoring method: Closeness, Degree, Edge Percolated Component (EPC), Maximal Clique Centrality (MCC), Maximum Neighborhood Component (MNC), and Radiality. Table S1. Samples sorted by K-means clusters C1 and C2. Table S2. Significantly upregulated DEGs in cluster 1 identified when comparing cluster 1 vs. cluster 2 using and FDR < 0.05 after post-hoc filtering performed on |log2(FC)| > 0.58. Table S3. Significantly upregulated DEGs in cluster 2 identified when comparing cluster 1 vs cluster 2 using and FDR < 0.05 after post-hoc filtering performed on |log2(FC)| > 0.58. Table S4. A gene set enrichment analysis was performed to identify gene-signature-based differences between C1 and C2 SNL samples. Table S5. Comparison of hub genes relative expression in C1, C2, and OSNA N0, with the NLN group. Table S6. Comparison of relative expression of DEGs in C1 and C2 with the NLN group. Table S7. Comparison of the relative expression of genes shared by the Oncomine™ Immune Response Research Assay panel and the normal LN dataset between the OSNA N0 and the NLN group. Table S8. Ratio between the normalised expression levels of CD28 and CTLA-4 genes in the NLN, OSNA N0, C1, and C2 groups. Table S9. Estimated absolute proportions of different cell populations using CIBERSORTx in OSNA SLNs homogenised samples profiled by RNA-Seq. Table S10. Comparison of immune cell populations between clusters C1 and C2.
