# Supplementary material for: Two Different Immune Profiles Are Identified in Sentinel Lymph Nodes of Early-Stage Breast Cancer
Source: Cancers (Basel). 2024 Aug 19;16(16):2881. doi: 10.3390/cancers16162881 (PMC11352239; doi:10.3390/cancers16162881)
Supplement: Supplementary file 1 [file cancers-16-02881-s001.zip › 2024.07.18_cancers_Supplementary Materials.pdf]

Supplementary Materials

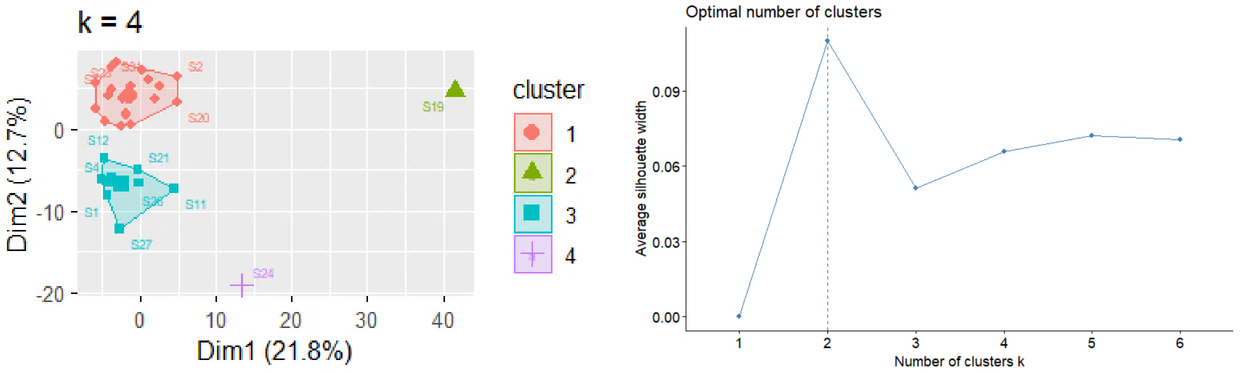

(a) (b)

Figure S1. (a) Visual assessment of the K-means clustering results when K=4. (b) Average silhouette method for different values of k computed after excluding the outlier samples S19 and S24. Results show that 2 clusters maximize the average silhouette width values.

Table S1. Samples sorted by K-means clusters C1 and C2.

**Sample**	**ONSA classification (1)**	**Kmeans Clusters**
S1	N0	C1
S2	N0	C2
S3	Nmi	C2
S4	N0	C1
S5	Nma	C1
S6	Nmi	C2
S7	N0	C2
S8	Nmi	C2
S9	Nmi	C2
S10	Nma	C2
S11	Nmi	C1
S12	N0	C1
S13	N0	C2
S14	N0	C2
S15	N0	C1
S16	Nmi	C2
S17	Nma	C2
S18	N0	C2
S20	Nma	C2
S21	Nmi	C1
S22	Nma	C2
S23	N0	C2
S25	Nma	C2
S26	Nma	C1
S27	N0	C1
S28	N0	C2

Sample	ONSA classification (1)	Kmeans Clusters
S29	N0	C1
S30	N0	C2
S31	N0	C2
s32	N0	C2

(1) - macrometastasis (pN1) as >5.10^3 copies/µL of CK19 mRNA, micrometastasis (pNmi) as 2.5.10^2 to 5.10^3 copies/ µL, and nonmetastasis (pN0) as <2.5.10^2 copies/ µL; n.d. – no data available.

Table S2. Significantly upregulated DEGs in cluster 1 identified when comparing cluster 1 vs cluster 2 using and FDR < 0.05 after post hoc filtering performed on |log2(FC)| > 0.58.

DEGs	Log2foldchange	Pvalue	FDR
IDO2	1.41	0.0001	0.0015
CD70	1.35	0.0007	0.0060
MADCAM1	1.16	0.0004	0.0034
CCL4	1.10	0.0056	0.0265
TNF	1.07	0.0009	0.0067
CCL3	1.03	0.0109	0.0442
MYC	1.01	0.0001	0.0009
CXCL1	0.93	0.0079	0.0350
CD80	0.86	0.0000	0.0000
PTPN6	0.78	0.0000	0.0000
CBLB	0.78	0.0000	0.0004
NCR3	0.73	0.0000	0.0001
KLF2	0.68	0.0001	0.0014
CD19	0.67	0.0001	0.0009
CD40	0.67	0.0000	0.0000
VEGFA	0.67	0.0024	0.0140
CD69	0.66	0.0075	0.0335
CD52	0.66	0.0000	0.0000
IL12A	0.66	0.0027	0.0152
GNLY	0.66	0.0033	0.0176
ID3	0.65	0.0034	0.0183
CD83	0.64	0.0048	0.0234

Table S3. Significantly upregulated DEGs in cluster 2 identified when comparing cluster 1 vs cluster 2 using and FDR < 0.05 after post hoc filtering performed on |log2(FC)| > 0.58.

DEGs	Log2foldchange	Pvalue	FDR
HLA-B	-3.79	0.0016	0.0106
LCN2	-3.57	0.0001	0.0009
FCGR3B	-2.22	0.0001	0.0009
CD44	-2.08	0.0038	0.0194
MRC1	-1.63	0.0000	0.0000
CCL18	-1.58	0.0000	0.0000
C1QB	-1.40	0.0000	0.0000
CD33	-1.14	0.0003	0.0026
CD209	-1.13	0.0000	0.0000

DEGs	Log2foldchange	Pvalue	FDR
CD163	-1.08	0.0000	0.0000
CXCR2	-1.06	0.0007	0.0055
C1QA	-1.04	0.0000	0.0000
FCGR3A	-1.01	0.0008	0.0061
CXCR6	-1.00	0.0000	0.0001
LST1	-0.94	0.0000	0.0000
PYGL	-0.93	0.0000	0.0003
CMKLR1	-0.88	0.0001	0.0010
KREMEN1	-0.87	0.0000	0.0000
IGF1R	-0.79	0.0019	0.0120
CCR2	-0.74	0.0000	0.0000
CSF1R	-0.73	0.0000	0.0000
VCAM1	-0.73	0.0000	0.0000
JCHAIN	-0.72	0.0107	0.0438
FASLG	-0.72	0.0001	0.0013
CX3CR1	-0.69	0.0111	0.0444
CEACAM1	-0.67	0.0023	0.0137
LAPTM5	-0.67	0.0000	0.0000
CD68	-0.61	0.0000	0.0001
CD14	-0.59	0.0006	0.0054

Table S4. Gene set enrichment analysis was performed to identify gene-signature-based differences between C1 and C2 SNL samples.

ID	Set Size	NES	P-adjust	Rank	Leading Edge	Core Enrichment
GSE8515_IL1_VS_IL6_4H_ STIM_ MAC_UP	21	2.290	3.93E-04	32	tags=48%, list=8%, signal=46%	CD70/EGR3/CCL4/TNF/CXCL1/ TNFSF9/CD80/CCL20/IL1B/CD83
GSE37532_TREG_VS_ TCONV_PPARG_ KO_CD4_TCELL_FROM_LN_DN	12	2.282	3.93E-04	32	tags=50%, list=8%, signal=47%	EGR3/EGR2/CCL4/PTPN6/CD40/ CD83
GSE14769_UNSTIM_VS_80MIN _LPS_ BMDM_DN	24	2.242	1.09E-03	32	tags=46%, list=8%, signal=45%	TNFSF14/EGR2/IFNB1/CCL4/ MYC/CXCL1/TNFSF9/CD69/ IL1B/ID3/ CD83
HALLMARK_TNFA_ SIGNALING_ VIA_NFKB	39	2.223	7.01E-05	36	tags=44%, list=9%, signal=44%	EGR3/EGR2/CCL4/TNF/MYC/ CXCL1/TNFSF9/CD80/IL1A/ KLF2/CCL20/VEGFA/CD69/IL1B/CD 83/IL12B/CCL2 IL12B/ CCL2
GSE8515_CTRL_VS_IL1_4H_STIM _ MAC_DN	12	2.201	1.81E-03	22	tags=50%, list=6%, signal=49%	CD70/EGR3/CCL4/TNF/CXCL1/ CCL20
DIRMEIER_LMP_ RESPONSE_EARLY	11	2.196	2.05E-03	32	tags=82%, list=8%, signal=77%	EGR3/EGR2/CCL4/TNF/CCL3/ MYC/TNFSF9/CD69/CD83
GSE2706_LPS_VS_R848_ AND_ LPS_ 8H_STIM_DC_DN	13	2.105	2.26E-02	55	tags=62%, list=14%, signal=54%	CD70/EGR3/TNF/TNFSF9/KLF2/ IL12A/ TARP/IFNG

ID	Set Size	NES	P-adjust	Rank	Leading Edge	Core Enrichment
NABA_SECRETED_ FACTORS	49	2.045	2.84E-02	62	tags=45%, list=16%, signal=43%	TNFSF14/IFNB1/CCL4/TNF/CCL3/ CXCL1/TNFSF9/IL1A/CCL20/ VEGFA/IL12A/IL1B/IL13/CCL17/ IL12B/CCL2/S100A9/CX3CL1/ CXCL13/IFNG/ IL15/TNFSF18
GSE19198_6H_VS_24H_ IL21_ TREATED_TCELL_UP	14	2.031	3.41E-02	39	tags=43%, list=10%, signal=40%	TNFSF14/EGR2/CD40/CD83/ CCL17/HLA-G
GSE40666_STAT1_KO_VS_ STAT4_ KO_CD8_TCELL_WITH_IFNA_ STIM_90MIN_DN	10	2.003	2.55E-02	25	tags=50%, list=7%, signal=48%	EGR3/EGR2/CCL4/TNF/VEGFA
Pro inflammatory	19	1.917	7.54E-03	55	tags=42%, list=14%, signal=38%	CCL4/TNF/CCL3/IL1A/CCL20/ IL1B/ CCL2/ IFNG
Inflammation-promoting	14	1.587	4.65E-02	63	tags=50%, list=17%, signal=43%	CD19/IL12A/GNLY/IL12B/CXCL13/ IFNG/ IRF1
CCR	68	1.439	4.65E-02	56	tags=28%, list=15%, signal=29%	TNFSF14/IFNB1/CCL4/TNF/CCL3/ CXCL1/G24TNFSF9/IL1A/CCL20/ IL12A/IL1B/IL13/CCL17/IL12B/ CCL2/CX3CL1/CXCL13/IFNG/IL15
HLA	16	-1.625	4.65E-02	42	tags=25%, list=11%, signal=23%	HLA-C/HLA-DQA2/HLA-A/HLA-B
CD141-positive Myeloid Dendritic cell - Lymph Node	42	-1.669	4.50E-02	68	tags=43%, list=18%, signal=40%	CXCL9/IGSF6/CCR1/TLR8/ HAVCR2/CD14/CD68/VCAM1/ CSF1R/CMKLR1/C1QA/CD163/ CD209/CD33/C1QB/CCL18/ MRC1/FCGR3B
Lymph Node-cd141-positive Myeloid Dendritic Cell	11	-1.742	4.50E-02	18	tags=27%, list=5%, signal=27%	C1QA/C1QB/HLA-B
Lymph Node-cd141-positive Myeloid Dendritic Cell	11	-1.749	4.96E-02	18	tags=27%, list=5%, signal=27%	C1QA/C1QB/HLA-B
macrophage - Lymph Node	33	-1.853	9.48E-03	93	tags=64%, list=24%, signal=53%	IL18/SLAMF8/TYROBP/CD4/ LILRB2/CXCL9/CCR1/TLR8/ CD14/CD68/VCAM1/CSF1R/ CMKLR1/FCGR3A/C1QA/CD163/ CD209/CD33/C1QB/CCL18/MRC1
Lymph Node-macrophage	11	-1.904	4.88E-03	18	tags=45%, list=5%, signal=45%	C1QA/CD163/C1QB/HLAA/ HLA-B
Small Intestine-neutrophil	11	-1.911	3.30E-03	18	tags=45%, list=5%, signal=45%	C1QA/CD163/C1QB/HLA-/ HLA-B
Lymph Node-macrophage	11	-1.911	3.30E-03	18	tags=45%, list=5%, signal=45%	C1QA/CD163/C1QB/HLA-/ HLA-B
Lymph Node Subcapsular Sinus macrophage - Lymph Node	34	-1.954	4.87E-03	80	tags=65%, list=21%, signal=56%	IL18/SLAMF8/CD4/LILRB2/ CXCL9/IGSF6/CCR1/TLR8/ HAVCR2/CD14/CD68/VCAM1/ CSF1R/CMKLR1/FCGR3A/C1QA/

ID	Set Size	NES	P-adjust	Rank	Leading Edge	Core Enrichment
						CD163/CD209/CD33/C1QB/ CCL18/MRC1
Bone Marrow-cd24 Neutrophil	16	-1.968	3.30E-03	23	tags=31%, list=6%, signal=31%	PYGL/HLA-A/CEACAM8/LCN2/ HLA-B
DELYS_THYROID_CANCER_ UP	29	-2.045	1.12E-02	63	tags=48%, list=17%, signal=44%	IGSF6/CCR1/ALOX15B/PTK7/ PRDM1/CSF1R/LST1/CX3CR1/ C1QA/C1QB/CCL18/MRC1/ CD44/LCN2

ID: unique identifier for each gene set; Set Size: the number of genes in the gene set; NES (Normalised Enrichment Score): the enrichment score representing the degree to which a gene set is overrepresented at the top (C1) or bottom (C2) of a ranked list of genes, normalized for the size of the gene set; P-adjust: the p-value indicating the statistical significance of the enrichment score, adjusted for multiple testing corrections using the Benjamini-Hochberg procedure; Rank: the rank at which the peak enrichment score occurs in the ranked list of genes; Leading Edge: The subset of genes contributing most to the enrichment score; Core Enrichment: The core set of genes driving the enrichment signal within the gene set.

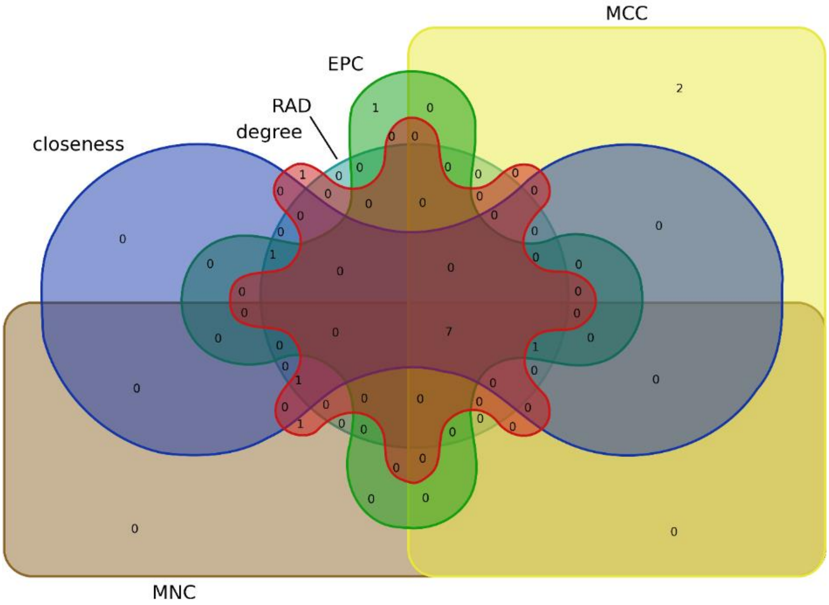

Figure S2. Hub genes were identified by overlapping the top DEGs of each scoring method: Closeness, Degree, Edge Percolated Component (EPC), Maximal Clique Centrality (MCC), Maximum Neighborhood Component (MNC) and Radiality. The Venn diagram was generated using the online tool available at http://bioinformatics.psb.ugent.be/webtools/Venn/.

Table S5. Comparison of hub genes relative expression in C1, C2, and OSNA N0, with the NLN group.

Hub gene	C1 P(1)	C2 P(1)	N0(1)	NLN P(1)	C1 vs. NLN(2)	C2 vs. NLN(2)	N0 vs. NLN(2)
CD80	41.5	32.8	37.2	27.5	2.07E-03*	5.85E-02	7.11E-03*
CD40	68.7	59.1	63.2	73.4	1.67E-01	8.47E-08*	2.17E-03*
TNF	53.4	43.3	48.9	31.6	1.39E-02*	1.74E-02*	2.91E-02*
CD163	68.9	82.1	79.5	59.3	2.02E-01	5.68E-05*	2.59E-03*
FCGR3A	29.1	38.8	34.8	42.7	1.71E-01	6.10E-01	3.58E-01
FCGR3B	10.8	18.3	14.1	19.4	1.86E-02*	8.03E-01	9.29E-02
CCR2	41.1	50.0	48.1	41.8	8.63E-01	2.29E-02*	1.55E-01

(1)P - mean of ranks for a particular group. (2) FDR - false discovery rate (* FDR < 0.05).

Table S6. Comparison of DEGs relative expression in C1 and C2 with the NLN group.

DEGs	C1 vs NLN FDR	C2 vs NLN FDR	C1 mean (rank)	C2 mean (rank)	NLN mean (rank)
IDO2	2.78E-01	4.59E-04	13.6	10.0	15.7
CD70	6.46E-02	4.87E-02	35.1	26.0	18.1
MADCAM1	2.41E-03	2.26E-05	17.7	13.3	8.0
TNF	1.39E-02	1.74E-02	53.4	43.3	31.6
MYC	8.34E-03	1.95E-01	80.4	71.3	66.1
CXCL1	9.40E-03	1.00E-01	32.8	24.2	18.2
CD80	2.07E-03	5.85E-02	41.5	32.8	27.5
PTPN6	8.39E-01	3.03E-04	88.3	82.3	88.7
CBLB	2.35E-02	1.37E-08	42.5	35.2	54.8
NCR3	2.03E-01	2.69E-01	41.8	33.9	37.4
KLF2	1.51E-04	3.15E-05	83.0	75.7	52.2
CD19	1.45E-02	1.69E-06	63.8	54.8	78.9
CD40	1.67E-01	8.47E-08	68.7	59.1	73.4
VEGFA	8.35E-01	1.23E-02	43.0	35.3	44.0
CD69	5.20E-01	5.85E-02	79.6	73.3	82.7
CD52	1.27E-01	6.14E-01	98.4	97.3	97.5
IL12A	2.83E-01	4.97E-01	14.7	12.6	13.3
GNLY	2.83E-01	6.95E-01	58.9	49.3	51.4
ID3	2.38E-01	1.50E-03	62.8	52.7	68.9
CD83	5.90E-02	1.36E-01	78.2	72.8	65.2
HLA-B	9.47E-04	7.90E-03	24.6	42.0	98.8
LCN2	3.81E-02	9.28E-01	7.0	11.8	12.1
FCGR3B	1.86E-02	8.03E-01	10.8	18.3	19.4
CD44	1.22E-12	1.08E-10	11.0	14.9	83.6
C1QB	6.84E-01	8.19E-04	88.7	95.5	89.8
CD33	1.53E-01	4.55E-01	27.2	38.7	35.6
CD209	1.45E-04	1.26E-09	85.9	93.2	40.6
CD163	2.02E-01	5.68E-05	68.9	82.1	59.3
CXCR2	1.90E-02	5.15E-05	19.0	25.6	11.1
C1QA	6.39E-02	9.62E-02	84.2	91.8	89.3
FCGR3A	1.71E-01	6.10E-01	29.1	38.8	42.7
CXCR6	1.54E-02	6.59E-02	23.7	32.7	40.9
LST1	2.79E-05	1.74E-04	42.6	54.6	68.1
PYGL	2.45E-03	5.44E-06	44.5	56.6	29.5
CMKLR1	4.82E-05	2.90E-05	22.8	30.3	46.7
KREMEN1	3.35E-01	4.14E-03	21.9	28.6	20.4
IGF1R	3.74E-06	5.61E-09	56.8	66.9	22.9
CCR2	8.63E-01	2.29E-02	41.1	50.0	41.8
CSF1R	6.58E-01	3.40E-04	69.5	79.6	67.6
VCAM1	1.68E-03	2.06E-09	87.6	91.5	80.8

DEGs	C1 vs NLN FDR	C2 vs NLN FDR	C1 mean (rank)	C2 mean (rank)	NLN mean (rank)
JCHAIN	7.61E-01	2.98E-01	95.4	97.0	95.8
FASLG	9.77E-01	1.12E-01	24.1	29.2	24.2
CX3CR1	4.94E-07	2.48E-12	5.6	7.4	16.6
CEACAM1	9.67E-02	7.03E-04	26.6	32.8	21.7
LAPTM5	9.47E-01	2.18E-03	96.5	98.0	96.5
CD68	1.01E-02	4.25E-08	91.6	94.5	86.1
CD14	4.03E-03	5.78E-07	83.6	88.6	62.7
% DEGs (FDR < 0.05)	48.9	63.8			

Table S7. Comparison of the relative expression of genes shared by the Oncomine™ Immune Response Research Assay panel and the normal LN dataset between the OSNA N0 and the NLN group. Only genes with significant differences (FDR<0.05) are shown.

Genes	N0 vs NLN FDR	N0 mean (rank)	NLN mean (rank)
MAGEC2	4.28E-285	1.58	2.11
CD44	2.61E-21	9.31	83.58
LCK	9.70E-16	17.14	84.05
HIF1A	7.45E-14	76.92	11.74
TGFB1	1.46E-12	85.76	61.53
NOS2	2.01E-12	1.84	10.47
HLA-F	5.91E-11	48.87	91.37
PTPRCAP	7.53E-11	27.30	70.21
CXCR3	1.72E-10	16.53	42.00
ITGAE	2.02E-10	27.91	68.47
HLA-DRA	2.46E-10	99.72	99.05
IRF9	2.46E-10	58.98	83.89
SELL	4.50E-10	55.79	90.21
JAML	8.48E-10	33.11	69.89
IL10RA	9.49E-10	86.25	74.05
HLA-DMB	9.55E-10	15.94	93.00
DDX58	1.51E-09	68.77	41.79
MMP9	2.50E-09	94.34	78.21
LAMP1	2.57E-09	57.66	78.00
TLR7	3.30E-09	55.53	26.16
CX3CR1	3.30E-09	6.48	16.63
CCL22	4.84E-09	79.87	27.79
TLR9	5.37E-09	58.54	19.05
TNFSF14	6.18E-09	6.22	19.05
TNFRSF14	1.29E-08	59.52	81.47
NOTCH3	1.29E-08	67.24	31.84
CD53	1.29E-08	66.71	94.53
NTN3	1.29E-08	10.53	4.68

Genes	N0 vs NLN FDR	N0 mean (rank)	NLN mean (rank)
FOXO1	1.29E-08	77.89	52.89
CYBB	1.41E-08	89.98	79.95
IGF1R	1.62E-08	60.90	22.95
IFI6	1.62E-08	78.77	37.95
ZAP70	1.93E-08	41.92	80.53
IFIT2	1.93E-08	59.67	32.16
HLA-DMA	1.97E-08	81.61	93.11
HLA-DQB2	2.26E-08	13.85	49.26
CD74	2.26E-08	95.13	99.68
BCL2L11	2.30E-08	33.72	53.16
PTEN	7.30E-08	79.84	52.58
TAGAP	9.91E-08	85.30	65.53
EIF2AK2	1.22E-07	63.16	36.00
CD209	1.74E-07	91.83	40.63
IL15	1.74E-07	21.35	40.63
TNFAIP8	1.88E-07	64.52	84.79
CD40LG	2.04E-07	72.98	42.37
ISG20	2.26E-07	54.95	86.05
CD4	2.29E-07	91.04	83.63
EGFR	2.57E-07	46.18	18.26
CSF2RB	2.65E-07	80.48	62.89
LY9	2.68E-07	40.20	58.32
AKT1	3.21E-07	55.72	72.68
CD3G	3.21E-07	86.07	62.37
HLA-DQA2	3.40E-07	10.43	58.21
CDKN3	4.93E-07	14.84	52.16
LRP1	5.37E-07	72.88	34.21
CCR6	7.61E-07	70.56	45.53
IFI27	7.64E-07	39.80	70.53
PSMB9	7.65E-07	76.28	89.11
MIF	7.65E-07	84.93	56.21
CD2	8.14E-07	90.03	82.58
CXCR5	8.14E-07	74.47	54.79
FCGR2B	1.23E-06	34.87	54.37
ICAM1	1.55E-06	79.11	59.68
KLRB1	1.96E-06	70.33	46.26
FOXP3	2.49E-06	44.82	24.11
IKZF1	2.73E-06	92.29	78.63
TNFRSF9	2.94E-06	58.55	32.47
CTSS	2.94E-06	93.04	88.74
LILRB1	2.95E-06	34.84	61.00
CD276	3.19E-06	18.52	37.63
BCL2	3.62E-06	64.98	47.05

Genes	N0 vs NLN FDR	N0 mean (rank)	NLN mean (rank)
NFATC1	3.74E-06	38.60	56.21
GRAP2	4.28E-06	69.38	50.95
IRS1	4.52E-06	38.34	15.95
BTLA	4.69E-06	74.08	53.68
PTPN11	5.17E-06	71.45	54.68
CCR7	6.76E-06	91.43	74.53
PIK3CA	7.24E-06	60.69	43.26
NRP1	8.48E-06	75.58	47.26
CA4	9.50E-06	31.38	8.89
HMBS	9.50E-06	25.21	40.16
NECTIN2	9.50E-06	24.18	41.26
CDK1	1.11E-05	29.29	62.84
RORC	1.28E-05	22.29	14.05
CD14	1.54E-05	88.13	62.74
GAGE12J	1.62E-05	7.48	0.79
CD1D	1.66E-05	23.21	31.00
CCL2	1.72E-05	26.15	70.00
CD27	1.74E-05	69.82	83.63
STAT3	2.21E-05	83.36	75.05
VCAM1	2.23E-05	90.51	80.79
CD48	2.44E-05	77.57	91.68
CMKLR1	2.78E-05	27.78	46.74
MAGEA10	2.90E-05	0.79	3.37
HLA-E	3.02E-05	98.27	96.84
ADGRE5	4.00E-05	81.51	72.32
BCL6	4.73E-05	46.41	67.42
CD68	5.20E-05	93.70	86.11
MAD2L1	5.47E-05	31.18	50.74
IDO1	5.47E-05	46.00	66.63
ITGA1	5.77E-05	54.24	34.42
LAMP3	6.37E-05	66.33	43.63
IKZF3	7.43E-05	81.96	57.16
IL12B	7.43E-05	13.83	8.32
CD79B	9.01E-05	77.12	90.42
KLF2	9.70E-05	77.98	52.16
IL2RB	1.51E-04	74.75	59.79
PDCD1LG2	1.55E-04	53.78	36.42
PTPRC	1.55E-04	85.76	94.58
OAS3	1.65E-04	51.50	34.89
POLR2A	1.65E-04	48.77	64.63
AXL	1.67E-04	44.54	27.32
GAGE1	1.74E-04	6.43	0.53
LAG3	2.13E-04	26.04	46.05

Genes	N0 vs NLN FDR	N0 mean (rank)	NLN mean (rank)
CXCR2	2.25E-04	23.36	11.05
MELK	2.28E-04	17.81	35.53
VTCN1	2.55E-04	2.37	4.37
FOXM1	2.70E-04	22.76	39.05
GATA3	2.70E-04	45.49	31.63
ITGAL	3.11E-04	71.86	81.11
CLEC4C	3.96E-04	40.46	23.00
CD19	4.31E-04	59.29	78.95
STAT4	5.14E-04	38.08	51.53
ABCF1	5.36E-04	70.28	62.21
CBLB	5.40E-04	38.55	54.79
CD28	5.60E-04	76.18	66.32
HLA-DRB1	5.60E-04	97.83	93.11
SIT1	5.60E-04	76.12	68.95
CXCL10	6.19E-04	45.82	66.53
CTAG2	6.20E-04	5.28	0.26
TNFSF13B	6.65E-04	60.12	44.21
MAGEA4	6.92E-04	1.32	7.58
IL21	6.99E-04	10.79	25.16
ID2	7.30E-04	61.64	74.58
PYGL	7.37E-04	53.01	29.47
HLA-G	7.65E-04	8.87	17.37
CCNB2	7.91E-04	30.66	55.16
IFIT1	9.13E-04	52.40	31.68
TNFSF10	1.00E-03	80.30	70.84
S100A9	1.04E-03	41.83	65.68
ZEB1	1.11E-03	57.17	46.79
BST2	1.23E-03	88.21	81.16
LST1	1.28E-03	50.86	68.05
MAPK14	1.28E-03	66.09	56.84
CD3E	1.49E-03	93.87	88.95
EBI3	1.52E-03	34.10	45.11
CD1C	1.52E-03	40.10	55.05
S100A8	1.52E-03	26.63	45.79
CCR1	1.68E-03	46.48	34.58
CCL17	1.69E-03	54.47	29.47
MAPK1	1.70E-03	66.79	58.79
TYROBP	1.70E-03	89.06	79.16
PMEL	1.70E-03	11.74	26.21
BRCA1	2.04E-03	20.35	33.11
BUB1	2.04E-03	29.80	47.89
CD40	2.17E-03	63.24	73.42
IL18	2.25E-03	64.52	75.79

Genes	N0 vs NLN FDR	N0 mean (rank)	NLN mean (rank)
NCAM1	2.38E-03	25.21	16.11
CD163	2.59E-03	79.51	59.26
IRF4	2.62E-03	62.75	53.63
GADD45GIP1	2.62E-03	66.81	57.84
AIF1	2.64E-03	74.18	84.32
IFNG	2.64E-03	14.85	27.11
HLA-B	2.64E-03	34.39	98.79
STAT5A	2.98E-03	60.84	70.26
IRF1	3.05E-03	69.39	79.00
M6PR	3.16E-03	87.76	84.21
SH2D1B	3.56E-03	20.69	13.58
TOP2A	3.62E-03	40.89	59.89
CD37	3.76E-03	96.30	92.84
ITGAM	4.01E-03	45.58	36.58
IL22	4.01E-03	3.06	1.32
ITGAX	4.01E-03	50.43	62.79
MADCAM1	4.01E-03	15.58	8.00
CD226	4.19E-03	47.37	38.32
IKZF2	4.24E-03	39.13	30.95
XAGE1B	4.27E-03	6.92	3.74
IL3RA	5.05E-03	54.16	45.26
CCR5	5.40E-03	49.41	40.95
LRG1	5.63E-03	18.14	10.42
TLR3	6.00E-03	21.07	12.16
HLA-DOB	7.11E-03	83.72	76.00
CD80	7.11E-03	37.20	27.47
ITGB1	7.21E-03	86.99	81.58
CCR4	7.72E-03	42.35	31.00
IFI35	8.54E-03	53.40	61.11
NT5E	8.85E-03	44.74	35.16
NKG7	1.04E-02	56.38	69.58
ENTPD1	1.04E-02	52.94	58.00
TFRC	1.37E-02	65.49	57.63
NCR1	1.50E-02	13.57	10.84
ALOX15B	1.50E-02	10.25	18.05
IL7	1.52E-02	31.05	42.05
CD79A	1.59E-02	95.94	92.95
MKI67	1.59E-02	28.22	42.16
ISG15	1.67E-02	61.12	46.89
TRIM29	1.67E-02	3.78	6.53
GZMK	1.67E-02	64.06	75.79
CD47	1.67E-02	83.13	77.42
IFITM2	1.78E-02	85.58	90.84

Genes	N0 vs NLN FDR	N0 mean (rank)	NLN mean (rank)
FUT4	1.95E-02	27.12	23.42
DGAT2	2.01E-02	41.07	23.05
IKZF4	2.02E-02	17.01	20.63
TDO2	2.03E-02	33.83	24.16
CX3CL1	2.07E-02	39.44	32.26
PRF1	2.11E-02	33.32	43.63
CD6	2.16E-02	57.89	67.47
CTLA4	2.40E-02	36.50	46.00
SSX2	2.56E-02	2.11	3.00
PVR	2.65E-02	28.29	22.37
CXCL1	2.70E-02	28.83	18.21
ICOS	2.75E-02	51.86	41.47
B3GAT1	2.76E-02	12.07	17.16
IL1B	2.78E-02	56.97	33.00
IL6	2.85E-02	20.87	29.32
TNF	2.91E-02	48.88	31.63
IFIH1	2.91E-02	51.60	44.47
CD63	3.03E-02	90.77	93.05
IL13	3.14E-02	7.98	4.95
HAVCR2	3.14E-02	57.89	48.84
KLRK1	3.21E-02	56.41	66.68
IDO2	3.33E-02	11.51	15.68
LYZ	3.39E-02	97.63	96.05
TUBB	3.41E-02	94.72	91.95
IL4	3.47E-02	6.43	9.37
CORO1A	3.55E-02	93.70	95.47
CEACAM1	3.66E-02	30.48	21.74
CXCL9	3.69E-02	69.64	85.53
TBX21	3.83E-02	25.86	30.47
GAGE10	3.88E-02	8.14	5.37
CD8B	4.04E-02	45.71	55.16
NFKBIA	4.11E-02	80.84	86.11
LMNA	4.11E-02	72.20	61.84
ARG1	4.20E-02	7.60	9.58
ID3	4.24E-02	56.40	68.89
EGR3	4.30E-02	56.58	36.63
FCER1G	4.43E-02	89.13	85.47
CD22	4.48E-02	76.84	84.95
KREMEN1	4.71E-02	27.14	20.37
FCGR1A	4.71E-02	17.75	35.05
CSF1R	4.78E-02	76.41	67.58

Table S8. Ratio between the normalized expression levels of CD28 and CTLA-4 genes in the NLN, OSNA N0, C1 and C2 groups.

	**Normalized expression level (mean)**			
**Genes**	**NLN**	**OSNA N0**	**C1**	**C2**
CD28	40.9	2474.2	2428.2385	2453.8
CTLA4	17.3	331.8	343.95035	334.2
CD28/CTLA4	2.4	7.5	7.1	7.3

Table S9. Estimated absolute proportions of different cell populations using CIBERSORTx in OSNA SLNs homogenised samples profiled by RNA-Seq. The results were determined using the custom signature matrix LM22.

Mixture	B cells naive	B cells memory	Plasma cells	T cells CD8	T cells CD4 naive	T cells CD4 memory resting	T cells CD4 memory activated	T cells follicular helper	T cells regulatory (Tregs)	T cells gamma delta	NK cells resting	NK cells activated	Monocytes	Macrophages M0	Macrophages M1	Macrophages M2	Dendritic cells resting	Dendritic cells activated	Mast cells resting	Mast cells activated	Eosinophils	Neutrophils	P-value
**S1**	0.8383	0	0.0162	0.1641	0.096	0.1537	0.057	0.0481	0.060	0	0	0.0712	0	0.1785	0	0.2645	0	0.0978	0	0.1491	0.1200	0	0
**S2**	0.4021	0.2222	0.0402	0.0896	0.013	0.2316	0.052	0	0.024	0	0.0281	0	0.0188	0.1158	0	0.4444	0	0.0827	0	0.0347	0	0	0
**S3**	0.5032	0	0	0.1511	0.202	0.3094	0.105	0	0.095	0	0.0748	0	0	0.1583	0	0.3659	0.0028	0.0869	0	0.0422	0	0	0
**S4**	0.9371	0	0.0661	0.1929	0.224	0.1003	0.066	0	0.051	0	0	0.0251	0	0.2029	0	0.2038	0.0119	0.0559	0	0.0456	0	0	0
**S5**	0.8124	0.2217	0	0.1108	0.012	0.1844	0.023	0.0465	0.046	0	0.0059	0	0	0.2837	0	0.2738	0	0.0985	0	0.0121	0	0	0
**S6**	0.4862	0.1109	0.001	0.2891	0.021	0.0188	0.195	0.0546	0.087	0	0.0307	0	0	0.2390	0.0011	0.4701	0	0.1410	0	0	0	0	0
**S7**	0.6341	0.1291	0.035	0.1577	0.172	0.1681	0.122	0	0.028	0	0.0620	0	0.0155	0.3041	0	0.1600	0	0.0597	0.0048	0	0	0	0
**S8**	0.4854	0.0669	0.023	0.1577	0.090	0.1826	0.037	0.0914	0.056	0.0393	0	0	0.0166	0.3333	0.0113	0.2188	0.0220	0.0953	0	0.2173	0	0.0025	0
**S9**	0.3601	0.1727	0	0.1260	0	0.3551	0.029	0	0.131	0	0.0083	0.0050	0.0389	0.1910	0.0138	0.2906	0	0.0236	0	0.0550	0	0.0250	0
**S10**	0.3915	0.1658	0.0326	0.1360	0.1411	0.2403	0.035	0.0242	0.117	0	0.0329	0	0.1071	0.2982	0	0.2142	0	0.1050	0	0.0080	0	0	0
**S11**	0.2822	0.1746	0	0.2040	0	0.2301	0	0.1000	0.120	0	0	0.0889	0.0434	0.1798	0.2703	0.0885	0.0029	0	0.2117	0	0	0	0

S28	S27	S26	S25	S23	S22	S21	S20	S18	S17	S16	S15	S14	S13	S12	Mixture
0.5435	0.6330	0.7214	0.4993	0.5488	0.6740	0.5030	0.5749	0.6480	0.5028	0.5000	0.8410	0.5538	0.5241	0.7680	**B cells naive**
0.1455	0	0	0.0652	0.2140	0.0110	0.2729	0	0.2110	0.1835	0.1779	0	0	0.1759	0.0763	**B cells memory**
0	0.006	0	0.040	0.044	0.017	0.014	0	0.0126	0.0407	0	0.0133	0.0152	0.0166	0	**Plasma cells**
0.0950	0.0677	0.1026	0.0906	0.0595	0.0923	0.0971	0.1153	0.0819	0.0742	0.2401	0.1176	0.1296	0.1333	0.0939	**T cells CD8**
0.0047	0.1269	0.0543	0.2172	0.1440	0.1614	0.0169	0.1034	0.1860	0	0	0	0.1054	0	0.0824	**T cells CD4 naive**
0.3725	0.1477	0.3219	0.3855	0.3075	0.3453	0.1482	0.2423	0.3390	0.3265	0.2790	0.3437	0.3132	0.2470	0.1533	**T cells CD4 memory resting**
0.040	0.007	0.031	0.061	0.050	0.034	0.052	0.069	0.029	0.049	0.051	0.000	0.034	0.011	0.007	**T cells CD4 memory activated**
0	0.0571	0.0055	0	0	0.0244	0.0236	0.0629	0	0	0.0037	0.0201	0	0	0.0543	**T cells follicular helper**
0.078	0.155	0.113	0.049	0.041	0.079	0.114	0.039	0.013	0.095	0.164	0.110	0.147	0.077	0.223	**T cells regulatory (Tregs)**
0.0195	0	0	0	0	0	0	0	0	0	0	0	0	0	0.0089	**T cells gamma delta**
0.0057	0.0325	0.0249	0.0281	0.0798	0	0.0463	0.0131	0	0.0171	0.0164	0.0286	0.0290	0	0	**NK cells resting**
0	0.0076	0	0	0	0.0096	0	0	0	0	0	0.0011	0	0.0098	0.0054	**NK cells activated**
0	0.0384	0.0367	0.0230	0.0485	0.0497	0	0.0719	0	0.0427	0.0324	0.0247	0.0927	0.0458	0	**Monocytes**
0.2988	0.1789	0.1346	0.2680	0.2994	0.2604	0.4098	0.2765	0.3413	0.2978	0.2767	0.3106	0.2582	0.2320	0.3423	**Macrophages M0**
0.0086	0.0050	0	0.0019	0	0.0012	0.0071	0.0096	0.0069	0.0314	0.0118	0	0.0048	0.0219	0.0031	**Macrophages M1**
0.3564	0.1899	0.1628	0.1619	0.2335	0.2054	0.2605	0.1834	0.2332	0.2032	0.2757	0.1790	0.1976	0.3125	0.3519	**Macrophages M2**
0	0.0989	0.0476	0	0	0	0	0	0	0	0	0.0126	0	0	0	**Dendritic cells resting**
0.0830	0.0821	0.1200	0.0619	0.0721	0.1145	0.0429	0.0901	0.0822	0.0117	0.0854	0.1520	0.0551	0.0199	0.0680	**Dendritic cells activated**
0.0142	0	0	0	0	0	0	0	0	0	0	0	0	0	0	**Mast cells resting**
0.0780	0.2261	0.0181	0.0286	0.0039	0.0424	0.0357	0.0392	0.0206	0.0154	0.0088	0.0610	0.0109	0.0196	0.0790	**Mast cells activated**
0	0	0	0	0	0	0	0	0	0	0	0	0	0	0	**Eosinophils**
0	0	0	0	0	0	0	0.0018	0	0	0	0	0	0	0	**Neutrophils**
0	0	0	0	0	0	0	0	0	0	0	0	0	0	0	**P-value**

Mixture	S29	S30	S31	S32
B cells naive	0.7408	0.5747	0.5074	0.6135
B cells memory	0.0076	0.0759	0.1612	0.1532
Plasma cells	0	0	0.0341	0.0213
T cells CD8	0.1346	0.1001	0.0494	0.1007
T cells CD4 naive	0.1550	0.2654	0.1047	0.0177
T cells CD4 memory resting	0.3177	0.1646	0.4651	0.1815
T cells CD4 memory activated	0.073	0.030	0.071	0.054
T cells follicular helper	0.0319	0.0331	0	0.0957
T cells regulatory (Tregs)	0.022	0.109	0.009	0.065
T cells gamma delta	0	0	0	0.0586
NK cells resting	0.0405	0.0305	0.0606	0
NK cells activated	0	0	0	0
Monocytes	0.0216	0	0.0326	0.0837
Macrophages M0	0.0901	0.3420	0.2564	0.2788
Macrophages M1	0	0.0054	0	0.0120
Macrophages M2	0.2535	0.4236	0.2701	0.2143
Dendritic cells resting	0.0879	0	0	0
Dendritic cells activated	0.0800	0.0775	0.0944	0.0703
Mast cells resting	0	0	0	0.0149
Mast cells activated	0.0451	0	0	0.0227
Eosinophils	0	0	0	0
Neutrophils	0	0	0	0
P-value	0	0	0	0

Table S10. Comparison of immune cell populations between clusters C1 and C2

CIBERSORT results(1)	C1 N = 10		C2 N = 20		P-values C1 vs C2
	mean	sd	mean	sd	p-value
B.cells.naive	0.708	0.192	0.526	0.083	0.016*(3)
T.cells.CD4.memory.resting	0.210	0.088	0.274	0.100	0.099(2)
T.cells.regulatory..Tregs	0.101	0.060	0.075	0.044	0.183(2)
Dendritic.cells.activated	0.080	0.042	0.076	0.031	0.766(2)
total.T.cells(7)	0.588	0.093	0.653	0.105	0.1080(2)
total.B.cells(7)	0.783	0.160	0.648	0.094	0.0302*(3)
DC total(7)	0.106	0.062	0.077	0.033	0.1896(3)
Lymphocyte.total(6)	1.409	0.132	1.329	0.119	0.105(2)

CIBERSORT results(1)	C1 N = 10	C2 N = 20	P-values C1 vs C2
	median	median	
B.cells.memory	0.004	0.149	0.198(4)
Plasma.cells	0.003	0.017	0.143(4)
T.cells.CD8	0.114	0.108	0.502(4)
T.cells.CD4.naive	0.068	0.104	0.522(4)
T.cells.CD4.memory.activated	0.027	0.049	0.12(4)
T.cells.follicular.helper	0.039	0.000	0.041*(4)
T.cells.gamma.delta	0.000	0.000	0.655(4)
NK.cells.resting	0.015	0.023	0.518(4)
NK.cells.activated	0.003	0.000	0.012*(4)
Monocytes	0.011	0.033	0.117(4)
Macrophages.M0	0.191	0.277	0.373(4)

CIBERSORT results[1]	C1<br>N = 10	C2<br>N = 20	P-values<br>C1 vs C2
**Macrophages.M1**	0.000	0.005	0.166[4]
**Macrophages.M2**	0.229	0.233	0.231[4]
**Dendritic.cells.resting**	0.007	0.000	0.003*[4]
**Mast.cells.resting**	0.000	0.000	0.823[4]
**Mast.cells.activated**	0.045	0.020	0.061
**Eosinophils**	0.000	0.000	0.1791[4]
**Neutrophils**	0.000	0.000	0.2206[4]
**NK.total**	0.035	0.023	0.1405[4]
**Macrophages.total**	0.466	0.529	0.2617[4]
**Mast.total**	0.053	0.020	0.0114*[4]
**T cells CD4 memory Activated to T cells CD4 memory Resting ratio**	0.111	0.160	0.391[4]
**NK cells Activated to NK cells Resting ratio**	0.136	0.005	0.1081[4]
**M2 to M1 ratio**	1710.000	59.900	0.2811[4]
**DC Activated to DC Resting ratio**	8.400	748.000	0.024*[4]
**Mast Activated to Mast Resting ratio**	453.500	131.500	0.0328*[4]
**Neutrophils-to-Lymphocyte ratio (NLR)**	0.000	0.000	0.0784[4]
**Monocyte-to- Lymphocyte ratio (MLR)**	0.007	0.023	0.0823[4]
**Lymphocyte to Monocyte ratio (LMR)**	6385.250	44.000	0.0823[4]

[1]Estimated absolute scores. SD—Standard deviation; * $p < 0.05$. [2] Student's t-Test (variances are equal); [3]Student's t-Test (variances are not equal, Welch correction was used); [4]Wilcoxon rank-sum (Mann–Whitney U) test. [5]For Eosinophils and Neutrophils, Shapiro-Wilk test was not performed as it requires at least 3 unique values. [6]Total lymphocyte fraction was calculated as a sum of all B cell, all T cell and all NK cell subpopulations. [7]Total fraction calculated as a sum of all subpopulations.
